# Meta-Analysis of *NOS3 G894T* Polymorphisms with Air Pollution on the Risk of Ischemic Heart Disease Worldwide

**DOI:** 10.3390/toxics6030044

**Published:** 2018-08-01

**Authors:** Robin Johns, Zhao-Feng Chen, Lufei Young, Flordelis Delacruz, Nien-Tzu Chang, Chong Ho Yu, S. Pamela K. Shiao

**Affiliations:** 1 College of Nursing, Augusta University, 987 St. Sebastian Way, EC 4505, Augusta, GA 30912, USA; rjohns@augusta.edu (R.J.); luyoung@augusta.edu (L.Y.); 2 Department of Nursing, Yuanpei University of Medical Technology No. 306, Yuanpei Street, 30015 Hsinchu, Taiwan; ivan.chen1966@gmail.com; 3 Department of Nursing, National University, San Diego, CA 92127, USA; delacruzflordelis@yahoo.com; 4 School of Nursing, National Taiwan University, Taipei 10051, Taiwan; ntchang@ntu.edu.tw; 5 Department of Psychology, Azusa Pacific University, Azusa, CA 91702, USA; cyu@apu.edu; 6 Medical College of Georgia, Augusta University, Augusta, GA 30912, USA

**Keywords:** meta-analysis, *NOS3* G894T gene, ischemic heart disease, cardiovascular disease

## Abstract

The purpose of this updated meta-analysis was to investigate the effect of nitric oxide synthase-3 (*NOS3)* G894T polymorphisms, air pollution and their interaction on ischemic heart disease (IHD) risk across populations worldwide. Recursive partition trees, nonlinear association curve fit and geographic information system maps were incorporated to verify results of conventional pooled analyses for sources of heterogeneity. Results from 61 studies (16,219 cases, 12,222 controls) revealed a significant increased relative risk (RR) of IHD associated with *NOS3* 894 polymorphisms TT (RR = 1.44) and GT (RR = 1.37). Subgroup analysis revealed that the TT polymorphism genotype had significantly increased risk of IHD in Caucasian, East Asian, South Asian, and Middle Eastern populations (all *p* < 0.05). It is important to point out that many countries demonstrated an average risk of greater than two, which identifies the *NOS3* 894 TT polymorphism as a potential causal factor and biological marker of IHD, based on criteria for strong evidence used in international consensus panels. These 10 countries include Ukraine, the United Kingdom, Brazil, Chile, Japan, South Korea, India, Iran, Egypt and Morocco. For these countries with elevated risk (RR > 2) from the *NOS3* 894 TT polymorphism, meta-predictive analysis demonstrated an increasing trend in air pollution association with increased *NOS3* 894 polymorphisms. Further studies are needed to explore the complexity of the associations among *NOS3* gene polymorphisms per population stratifications within countries, detailed air pollution data for added specificity for geographic location across time, and disease risk.

## 1. Introduction

Ischemic heart disease (IHD) is the leading cause of death and a major economic burden worldwide. It is the cause of over 30% of total annual deaths [1,2,3,4] and constitutes 17% of overall national health expenditure in the United States (U.S.) [5]. It is projected that by 2035, IHD will affect 131.2 million Americans (45 percent of the total U.S. population) with an annual cost of $1.1 trillion, resulting in a crushing impact on the nation’s financial and health care systems [6]. Many epidemiological studies have revealed substantial evidence of genetic components (ranging from 20–60%) that contribute toward the progression of IHD; however, etiologic pathways associated with gene polymorphisms and the risk of IHD remain unclear [7,8].

Endothelial-derived nitric oxide (NO) is a powerful vasodilator with known vasoprotective effects, and its physiologic functions are as follows: (1) vascular relaxation; (2) platelet suppression; (3) decreased leucocyte adhesion to the vascular endothelium; (4) reduced smooth muscle cell proliferation and migration [9,10,11,12]; and (5) reduction of atherogenic low density lipoprotein oxidation [12]. NO is synthesized from L-arginine by nitric oxide synthase-3 (NOS3), a protein coding gene located on chromosome 7q35–36 [11]. Variations in this gene are associated with susceptibility to coronary spasm and are known to have a major effect in the development of atherothrombogenesis [13]. The single nucleotide polymorphism (SNP) *NOS3 894GT* located in exon 7 (also known as *Glu298Asp,* rs1799983) is a genetic marker that has been specifically linked to an increased risk of IHD, hypertension, coronary spasms, and stent re-stenosis [8,14,15]. The *NOS3 894GT* SNP represents a guanine (G)/thymine (T) substitution at position 894 on exon 7 leading to a change from glutamate to aspartate at position 298; rs1799983 [11]. NOS3 activity is reduced in the presence of the T allele for *NOS3* G894T polymorphisms [16], with previous meta-analyses confirming the association of *NOS3* G894T with the development of IHD among various ethnicities and disease subgroups [12,17,18,19,20,21].

Likewise, there are several epidemiologic studies suggesting the incidence of IHD morbidity and mortality may be related to exposure to ambient pollutants that trigger the development of IHD [22,23,24,25,26,27,28,29]. Many studies examining gene-environment interactions have found air pollution may be involved in modifying genetic variants and associated disease risk [30,31,32,33,34,35,36,37,38,39,40,41,42] as well as levels of inflammatory markers and subsequent risk of myocardial infarction (MI) [43]. Thus, there is evidence that associations of air pollution with gene markers for cardiovascular risk are mediated by oxidative stress pathways. Inhalation of airborne particulate matter (PM) inhibits both nitric oxide synthase activity and nitric oxide release, resulting in vasoconstriction [38,41]. Oxidative stress induced by PM-dependent reactive oxygen species (ROS) production affects vascular function by disrupting endothelial function; and PM exposure has been demonstrated to potentiate hypertension through NOS-dependent ROS generation [42]. In addition, investigation of associations of air pollution exposure with blood pressure and heart rate variability revealed both are modified by gene polymorphisms in the oxidative stress pathways, including *NOS3* [37]. Because *NOS3* affects metabolism in the urea cycle of the methylation pathway, which is critical for preventing systemic inflammation as an epigenetic risk factor for heart health, it is important to explore associations among *NOS3* 894 gene polymorphisms, air pollution, and IHD [44,45,46,47].

Therefore, the purpose of this updated meta-analysis is to examine the association of *NOS3* 894 polymorphisms as risk factors for IHD, using meta-predictive analytics to investigate the source of heterogeneity, including air pollution for IHD susceptibility. This study is the most comprehensive meta-analysis to date on the impact of *NOS3* 894 on IHD. The gaps in knowledge were filled that were not addressed in previously published meta-analyses which focused only on sub-populations (Asians) and sub-types of IHD.

## 2. Materials and Methods 

### 2.1. Search Strategy and Selection Criteria

Online databases of PubMed, PubMed Central and Google Scholar were searched for all available studies from 1998 (publication year of first related study) through September 2017 following the guidelines for preferred reporting of items for meta-analysis of observational studies [48,49,50]. The search was conducted using the following keywords and subject terms: *NOS3* 894, ischemic heart disease, coronary heart disease, meta-analysis, case-control, and was limited to human studies. In addition, previous meta-analyses and review papers were used to cross reference and trace back to all original studies. Databases were searched repeatedly every three months for a total of four different times to identify any new papers. Two investigators were responsible for conducting the literature search and recording data. Studies published in other languages or from other countries were included, but only if they included both an abstract in English and tables clearly listing genotype allele counts. Studies lacking genotypes for case and controls or appropriate genotype allele counts were excluded.

**Figure 1:**
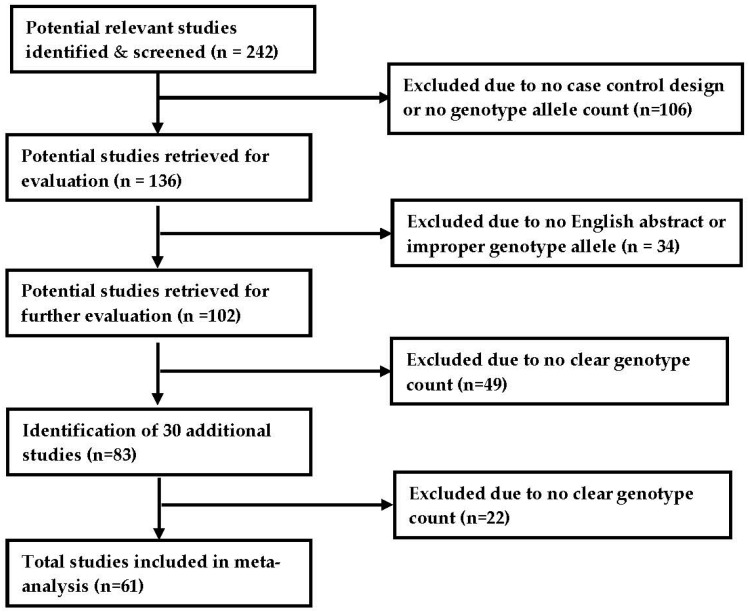
Progression of the selection of studies for the meta-analysis.

Two hundred and forty-two relevant studies were initially identified, with 106 subsequently excluded because they did not have genotypes for case and controls and/or genotype allele counts. The 136 remaining studies were retrieved for evaluation. Of those, 34 studies were further excluded due to no English abstract and/or no appropriate genotype allele counts. The remaining 102 studies were retrieved for further evaluation, of which 49 studies were further eliminated due to no clear genotype counts. Further searches resulted in the identification of eight additional usable studies from a list of 30 studies. Thus, a total of 61 studies were included in the final meta-analysis (Figure 1).

### 2.2. Characteristics of Included Studies

Study populations were drawn from Australia, Europe, Central America, South America, Asia, the Middle East, and Africa. The majority of populations from these studies were Caucasian (25) and East Asian (18) studies, followed by South Asian (six), African (six), Middle Eastern (four), and Hispanic (two) (Supplementary Table S1). The air pollution markers we used is per country data, because the source data from each original study were reported per countries and missing specific locations within the country. While we attempted to examine specific locations for each country per study, many studies did not report specific locations within each country. Further scale for geographic location per country would yield missing data and insufficient numbers for meta-analysis. Air-quality data was entered for all countries. Specifically, we verified the most current and complete air-pollution data including the death rates from air pollution (death rates per million, Level 1: <50, Level 2: 51–100, Level 3: 101–250, Level 4: 251–400, Level 5: ≥401) [51,52,53]. Current scales on air pollution data were used to further verify these levels [54,55,56], and the most complete and current data on air pollution was used for the analyses. There was only one study (Ireland) with a Level 1 air pollution death rate (AP death) level and one study (Ukraine) with a Level 5; therefore, Level 1 was merged with Level 2 and Level 5 was merged with Level 4 for the final analysis.

### 2.3. Quality Assessment

Data including genotype allele counts and quality scores were checked for accuracy by three team members; for discrepancies, a consensus was reached by involving a fourth team member based on the criteria for quality in published meta-analyses (PRISMA) and other observational studies [30,49,50]. The quality scores for included studies ranged from 10 to 25 (possible score range of 0–29). For the representative DNA analysis, Hardy Weinberg Equilibrium (HWE) analyses were checked for all studies, which were developed to assess the distribution equilibrium for evolutionary mechanisms associated with population genetics [57,58]. Characteristics of all included original studies on *NOS3* 894 genotypes are reported in Supplementary Table S1.

### 2.4. Data Synthesis and Analysis

Genotype allele counts, along with all other data, were entered into Excel (Microsoft Corp, Redmond, WA, USA), and pooled using Stats Direct Version 3.1.4 (Cheshire, UK). Pooled risk ratios (RR) for *NOS3* 894 genotypes for cases and controls and 95% confidence intervals (CI) were calculated for the associations of polymorphisms and IHD with heterogeneity tested before selection of an association model. Pooled RRs are preferred to odds ratios (OR) according to related consensus reports for the field of study [30,59,60]; they have been demonstrated to be more robust and better for comparison due to standardization of the denominator as opposed to using only one genotype as the denominator [30,31,32,33,34,35,59,60,61,62,63]. We used total counts of all three *NOS3* 894 genotypes (homozygous TT, heterozygous GT, and wild-type GG genotypes) as the denominators to compute standardized ratios for RRs. Thus, a pooled RR of greater than one represents an increased risk for IHD among gene variations, whereas a pooled RR less than one indicates a protective effect (favoring the case or the IHD group). Significant findings were identified at a value of *p* < 0.05.

Additionally, because the data presented heterogeneity with some regional differences on polymorphism rates and risks, the JMP 13 Pro program (SAS Institute, 2016) was used to generate geographic information system (GIS) maps to depict global gene polymorphism patterns and IHD risks, allowing visual identification of geographic patterns [64]. JMP is a statistical program for data visualization, exploratory data analysis, and data mining, and these features are instrumental to this study. Furthermore, meta-prediction analysis using JMP 13 Pro was incorporated to examine how AP death (independent variable) decisively split the dependent variable data (polymorphism genotype frequencies and IHD risks) [30]. Meta-prediction methods integrated multiple statistical models for triangulation purposes [30,65]. Through triangulation, sources of heterogeneity were explored and identified for divergent *NOS3* 894 polymorphism rates and IHD risk; GIS maps were generated to manage the geospatial data set and help associate regional patterns of polymorphisms and IHD risk with AP death by country [30,64]. Recursive partition analysis using JMP 13 Pro was incorporated to create a decision tree that classified groups of population (*NOS3* 894 polymorphism rates in cases and control groups and risks) by splitting data into subgroups based on levels of AP death [30,33,66]. We used AP death instead of air pollution or air quality index because without indicating the outcome (death), air quality index alone could be misleading. Prior research had suggested that PM can endanger public health when varying weather conditions are taken into account [67]. In other words, the risks of respiratory and circulatory system diseases increase when the effects of certain weather conditions are present. However, the weather characteristics associated with PM have not been thoroughly studied [68], and thus we chose an outcome-based index (AP death) instead.

To provide consistency with other meta-analyses and allow for easy comparison, we also employed a conventional multiple comparison procedure (Tukey’s test) [69] to examine whether meta-predictive data analytics (recursive partition trees) and Tukey’s tests concurred with each other when examining the interaction between gene mutation and air pollution (AP), and its prediction on IHD risk [33]. The advantage of meta-prediction over conventional statistical methods (meta-regression) is that these analytics (e.g., recursive partition trees) are useful in verifying results by cross validation [70,71,72] and enhance sensitivity and specificity [73], and thus may yield more accurate meta-predictive results [74]. Akaike’s Information Criterion (AIC) was employed for selecting the optimal number of subgroups, from which the model yielding the smallest AIC value is utilized [75] Finally, we also used a nonlinear fit curve to examine the associations between AP death and the polymorphism genotype percentage and IHD risks. By integrating data from diverse sources, advanced techniques including recursive partition trees and nonlinear fit are able to predict more accurately and precisely, thus strengthening our analysis. While meta-regression is used commonly for advanced meta-analysis for meta-prediction [63], it is important to point out that regression analysis, as a linear model, is unable to detect nonlinear patterns. In this study, we performed meta-prediction using recursive partition trees, nonlinear fit, and heat maps for data visualization to reveal nonlinear patterns. Further, it is well known that regression based on R^2^ tends to yield a complex and over-fitted model because *R*^2^ always goes up with additional predictors. On the other hand, AIC or Akaike’s information criterion correction (AICc) does not necessarily change with the addition of variables. Rather, it varies based upon the composition of the predictors; thus, it is more likely to yield an optimal model [76].

## 3. Results

### 3.1. Pooled Meta-Analysis

Pooled analysis of *NOS3* genotypes from 61 studies with genotypes for case and controls was used to identify the association of *NOS3* 894 gene variations with IHD in global populations. The analysis included a total of 16,219 IHD cases and 12,222 controls (Table 1). The GG wild genotype (53.1% cases and 60.8% controls) presented as the most frequent genotype across all populations and was found to be protective against IHD for all racial-ethnic groups (RR = 0.92, *p* < 0.0001). The heterozygous GT polymorphism genotype (37.4% cases and 32.4% controls) was associated with an increased risk of IHD for all groups (RR = 1.37, *p* < 0.0001). The homozygous TT polymorphism genotype (9.5% cases and 6.2% controls) presented the least frequently, and, like the GT polymorphism, was associated with an increased risk of IHD for all groups (RR = 1.44, *p* < 0.0001). Furthermore, the T allele was associated with an increased risk of IHD for all groups combined (RR = 1.18, *p* < 0.0001). In contrast, the G allele was found to be protective against IHD for all racial-ethnic groups (RR = 0.95, *p* < 0.0001). Homozygous TT polymorphism percent distributions were observed to be higher in Caucasians (10.2%) than in African (8.0%), Hispanic (4.4%), Middle Eastern (4.2%), South Asian (2.0%) and East Asian groups (0.7%), in the control groups as the basis for healthy populations. GT polymorphism percent distributions were observed to be higher in Caucasians (43.6%) than in African (38.2%), Hispanic (32.8%), Middle Eastern (30.6%), South Asian (24.4%) and East Asian groups (14.6%), in the control groups as the basis for healthy populations. Similar distinctive patterns were noted for the IHD cases across all of the racial-ethnic groups. Supplementary Figure S1 presents the percent homozygous TT and heterozygous GT genotypes for the countries included in the analyses. When visualizing forest plots for the meta-analysis, most studies presented both *NOS3* 894 TT and GT genotypes as risk genotypes (RR > 1) of IHD. Racial-ethnic subgroup analysis revealed that the homozygous TT genotype was associated with an increased risk of IHD in Caucasian (*p* = 0.0051), East Asian (*p* < 0.0001), South Asian (*p* = 0.0076), and Middle Eastern (*p* = 0.0003) populations. The heterozygous GT genotype was also associated with an increased risk of IHD for both East Asian (*p* < 0.0001) and South Asian (*p* = 0.0304) populations.

### 3.2. Subgroup Analysis

**Figure 2:**
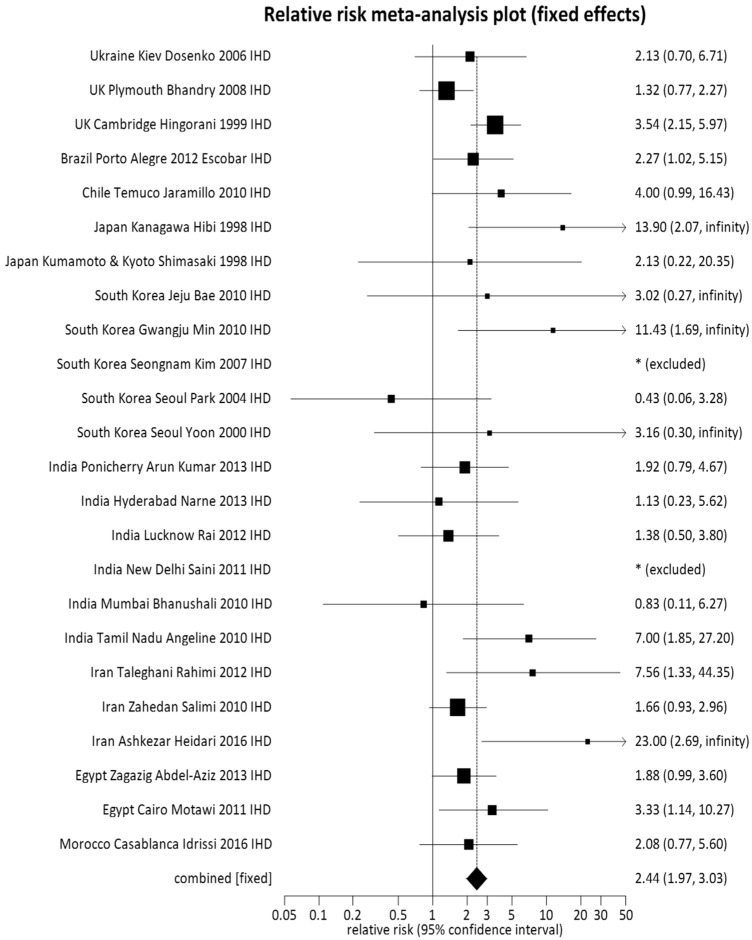
Forest plot for countries with pooled relative risk (RR) > 2 for the meta-analysis of the association between the *NOS3* TT genotype and ischemic heart disease (IHD). The midpoint = point effect estimate for each study. The area of the box = weight given to the study. The length of the line = 95% confidence interval for the effect estimate in an individual study. The width of the diamond = 95% confidence interval for the overall effect estimate. Note * (excluded): study excluded due to no TT genotypes reported for case/control groups.

Subgroup analyses were conducted to explore the significant heterogeneity across regions for the *NOS3* 894 TT genotype split by countries that demonstrated a risk type (RR > 1–2, RR > 2) and countries with a protective type (RR < 1) (Figure 2, Supplementary Table S2, Supplementary Figures S2 and S3). Subgroup analysis allowed for stratification of TT risk (TT risk greater than 2, TT risk between 1–2, and TT risk less than 1) and revealed 10 countries with a TT risk > 2: Ukraine, the United Kingdom, Brazil, Chile, Japan, South Korea, India, Iran, Egypt, and Morocco (Figure 2); nine countries with a TT risk > 1–2: Turkey, Greece, Italy, Finland, Germany, Ireland, Mexico, China, and Saudi Arabia (Supplementary Figure S1); and four countries with a TT risk < 1: Australia, The Netherlands, Poland, and Tunisia (Supplementary Figure S3). When visualizing forest plots by RR subgroups, it was revealed that Ukraine, the United Kingdom, Brazil, Chile, Japan, South Korea, India, Iran, Egypt and Morocco presented the *NOS3* 894 TT genotype as a potential causal factor of IHD [59,60,61,62] (Figure 2). In contrast, the *NOS3* 894 TT genotype was protective for Australia, the Netherlands, Poland and Tunisia (Supplementary Figure S3).

**Figure 3:**
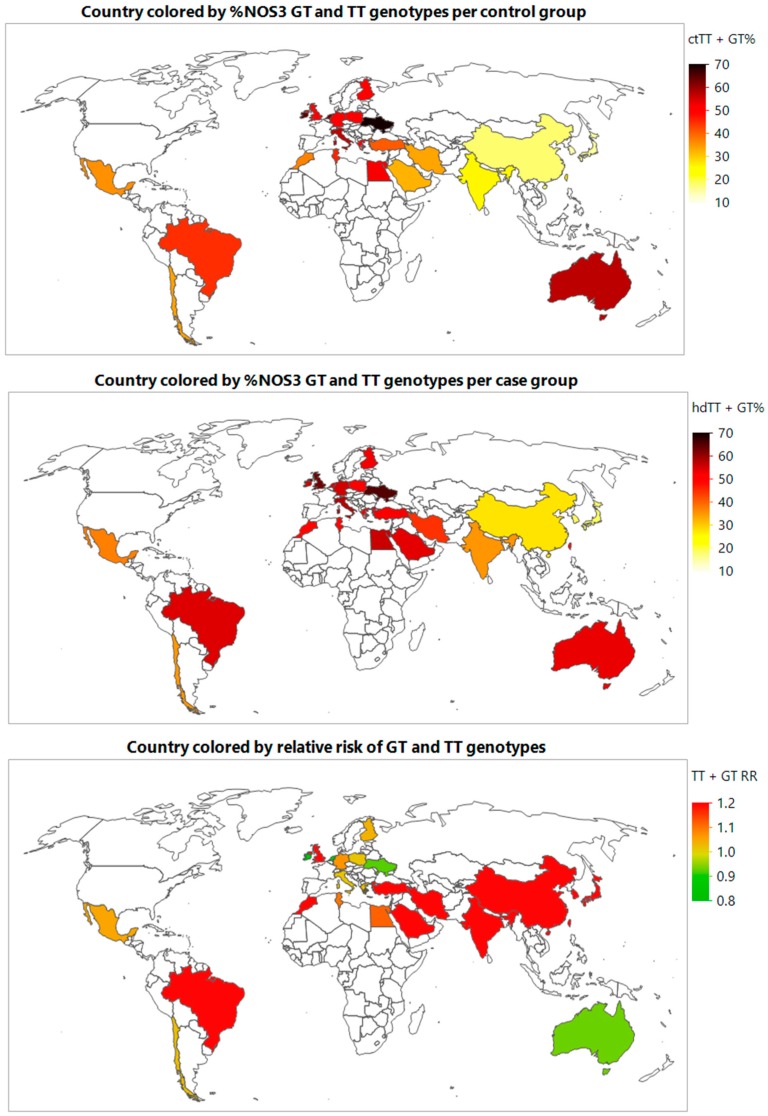
Geographical information map for % *NOS3* GT and TT genotypes per control and ischemic heart disease (IHD) groups, and IHD risk.

The incorporation of GIS maps allowed for further illustration of *NOS3* 894 polymorphism rates and IHD risk association and enabled visual detection of regional patterns. The GIS maps graphically depict the highest total percent of *NOS3* 894 TT and GT genotypes for both control and cases groups in Caucasian nations (Figure 3, Supplementary Figures S4 and S5). However, the highest risks of IHD from *NOS3* 894 TT and GT polymorphisms were in East Asian and South Asian nations (Figure 3, Supplementary Figures S4 and S5). For individual *NOS3* 894 TT and GT polymorphisms, similar patterns were also demonstrated (Supplementary Figures S4 and S5). RRs were presented using a chromatic color spectrum with the red color representing IHD risk and the green color representing protective effects. Regions with the highest risk of combined *NOS3* 894 TT and GT genotypes were observed as Asia and Africa, followed by South America, then Europe (Figure 3). A similar pattern was observed for both individual *NOS3* 894 TT and GT genotypes (Supplementary Figures S4 and S5).

### 3.3. Meta-Prediction

**Figure 4:**
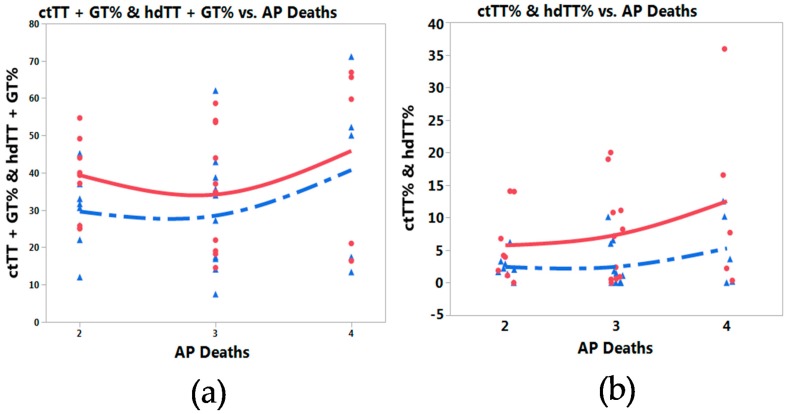
Nonlinear fit of *NOS3* G894T polymorphisms with death from air pollution bi-variate plots for countries with pooled RR > 2: (**a**) TT plus GT polymorphisms, (**b**) TT only (AP death: Death rates from air pollution, Levels per million: 2 = 51–100, 3 = 101–250, 4 = 251–400 and greater); case = red (solid line), control = blue (dotted line).

Meta-prediction analytics were incorporated to explore air pollution death rates for each country as possible contributing variables to *NOS3* 894 SNP percentages and risks for IHD. Partition tree analyses and Tukey’s tests were used to test both variables. For partition trees, Akaike’s information criterion correction (AICc) was used to select the best model with good fit to the truth with simplicity and parsimony [30,74]. The partition tree split the data into two groups by annual AP death rate levels (Levels 2 = 51–100, 3 = 101–250, 4 = 251–400 death/million populations). While no statistical significance was found, this analysis revealed a trend in the association among air pollution death rates, *NOS3* 894 polymorphisms and heart disease. Nonlinear association methods were used to explore the association between AP death and the *NOS3* percentage of polymorphisms (Supplementary Figure S6) for both groups with TT plus GT polymorphisms (Supplementary Figure S6a) and TT polymorphism only (Supplementary Figure S6b. With a change in air pollution death rates from Level 3 to Level 4 using Tukey’s tests, there was a upward trend in the percentage of *NOS3* 894 polymorphisms with an even more substantial increase in this trend for countries with a RR greater than 2 (Figure 4a for TT plus GT polymorphisms, Figure 4b for TT polymorphism only, bi-variate plots). For the 10 countries with a TT risk > 2, Brazil, India, and Morocco presented AP death Level 2; Chile, South Korea, Iran, and Egypt presented AP death Level 3; and Ukraine, United Kingdom, and Japan presented AP death Level 4 (Figure 4a,b).

## 4. Discussion

Our meta-analysis represents the most comprehensive study focusing on the association between *NOS3* 894 polymorphisms and the risk of IHD to date, integrating air pollution data to explore the source of heterogeneity across the world’s regions. We conducted meta-prediction analyses to examine the potential impact of air pollution on the link between *NOS3* polymorphisms and IHD risks. Although theoretically there is a potential risk of ecological fallacy, the use of global data was necessary because large-scale individual data is not available. Tools for monitoring the amount of pollutant absorbed by an individual with the individual’s biophysical characteristics and the space-time activities are in development, however the use of such are not prevalent across countries [77]. Although meta-regression is frequently used for meta-prediction in advanced meta-analysis [63], it is important to point out that as a linear model, regression analysis is unable to detect nonlinear patterns. Furthermore, critiques of linear modeling, including Pearson’s correlation and regression, reported issues being overly simplistic; nonlinear modeling is more appropriate when the underlying data structure is nonlinear, such as the relationship between air pollution and death [78,79,80]. Because AIC or AICc does not necessarily change with the addition of variables and instead varies based upon the composition of the predictors, it is more likely to yield an optimal model [74]. As such, the use of multiple meta-analytics was not only helpful to verify the results across the analytics, but also to visually detect regional geographical patterns.

*NOS3* affects metabolism in the urea cycle of the methylation pathway, critical for preventing systemic inflammation. Vascular effects of oxidative stress resulted from air pollution are mediated by NOS which is reduced in the presence of the *NOS3* 894 T polymorphisms [15] leading to endothelial dysfunction [79]. Thus, airborne exposure to ambient PM is a major cardiovascular health threat. In 2016, of the 4.1 million deaths attributed to PM2.5 exposure, most (58%) were IHD and stroke [81]. Because airborne PM has the capacity to generate oxygen free radicals, PM exposure may trigger a systemic inflammatory effect through oxidative stress mechanisms [80]. Changes in the expression of genes related to oxidative stress pathways have been shown to be differentially regulated by PM exposure [82]; thus, PM has the capacity to induce a pro-inflammatory phenotype consistent with vascular disease progression [83]. Because air pollution may compromise methylation pathways to increase systemic inflammation, high risk groups with polymorphisms in the methylation pathways are more vulnerable to develop health problems. Therefore, a better understanding of possible connections between environmental pollution, *NOS3* polymorphisms, and IHD can be useful in developing potential prevention strategies. Further studies are needed to examine the complexity of the associations among *NOS3* gene polymorphisms per population stratifications within the countries with accurate measurement of exposure to air pollution in relation to geographic location across time, and disease risk.

Our meta-predictive analysis demonstrated an upward trend in air pollution death rate level associated with the *NOS3* 894 TT homozygous genotype for countries with a RR greater than 2. While our findings were not significant, this trend is consistent with the emerging pattern of association of air pollution with gene polymorphisms and disease risk [30,31,32,33,34,35,36,37,38,39,40,41,42,43,44,45,46,47]. Previous studies have found significantly increased polymorphisms of the *MTHFR* gene with increased air pollution levels in various conditions including colorectal cancer, breast cancer, leukemia, Alzheimer’s disease, and hypertensive disorders during pregnancy (HDP) [30,31,33,34,35], and increased disease risk for HDP [35]. Although the *MTHFR* gene is known to be heat sensitive [84,85], there is no current evidence of heat sensitivity related to the *NOS3* mechanism; however, the trend of increased *NOS3* polymorphisms with increased air pollution for countries with higher risk (RR > 2) is consistent with each of these previous studies [30,31,33,34,35]. In the global context, Asian countries tended to have higher air pollution death rates in recent years but did not yet demonstrate an associated increase in *NOS3* polymorphisms; however, Asian countries presented with higher IHD risk in association with *NOS3* 894 polymorphisms.

Our analysis revealed significant associations between *NOS3* 894 TT and GT (polymorphisms with IHD risk across all racial-ethnic populations), whereas the common allele *NOS3* GG genotype was observed to be protective for all groups. The T allele was associated with an increased risk of IHD for all groups, and, consistent with reports from previous meta-analyses, suggests those carrying the *NOS3* 894 T allele may have a predisposed risk for IHD [12,17,18,19,20,21]. It is important to point out that upon subgroup analyses, 10 countries (Ukraine, the United Kingdom, Brazil, Chile, Japan, South Korea, India, Iran, Egypt and Morocco) demonstrated an average RR greater than 2, which presented the *NOS3* 894 TT polymorphism genotype as a potentially causal factor of IHD as a biological marker based on the criteria for strong evidence used in international consensus panels [59,60]. For these countries, meta-predictive analysis demonstrated an increasing trend in air pollution association with increased *NOS3* 894 polymorphisms.

Heterogeneity with regional differences on polymorphism rates and IHD risk was demonstrated by the data. Congruent with our study, others have also identified ethnicity as the source of heterogeneity in the association between *NOS3* 894 polymorphism and MI risk among Asians vs. non-Asians [18]. Mechanistic studies have shown the impact of *NOS3* 894 polymorphisms on IHD risks could undergo several potential pathways (e.g., reduced NO level, increased oxidative stress, impaired endothelial function, and increased vascular inflammation) in Asian populations [86,87]. There is also evidence demonstrating significant associations between *NOS3* 894 polymorphisms and two leading risk factors of IHD, hypertension and type 2 diabetes in Asians [88,89,90,91]. Additional factors including age, gender and PM concentration may also play a role in gene-environment interaction among Asians through oxidative stress pathways [92,93]. Additional studies including large scale sample size and samples from various race/ethnic groups are needed to explain the different mechanisms contributing the impact of *NOS3* 894 on IHD risk among various ethnic groups/regions.

## 5. Conclusions

The results of our comprehensive meta-predictive analysis demonstrated differences in the rate of *NOS3* 894 polymorphisms and associated IHD risk across global populations. This analysis revealed a trend in the association between increased air pollution associated death rates with increased *NOS3* 894 polymorphisms. These findings provide novel insight regarding the association between *NOS3* 894 polymorphisms, air pollution, and IHD risk. Both homozygous TT and heterozygous GT genotypes of *NOS3* 894 in Caucasian populations were found to be higher than in other populations including African, Hispanic, Middle Eastern, South Asian and East Asian. However, both homozygous and heterozygous *NOS3* 894 polymorphism-associated IHD risks are higher in Asian nations when compared to other nations. Future studies exploring epigenetic factors in association with *NOS3* 894 polymorphisms and IHD and prevention strategies for IHD are needed.

## Figures and Tables

**Table 1:** Pooled meta-analysis: *NOS3* G894T genotypes and risk for ischemic heart disease (IHD).

Genotype	Cases	Controls		Tests of Association	
(Number of Studies)	*n* = 16,219 (%)	*n* = 12,222 (%)	Model	RR (95% CI)	*p*
TT (61)	1551 (9.56)	759 (6.21)	Random	1.44 (1.23, 1.67)	0.0001
Caucasian (25)	1230 (12.30)	593 (10.21)	Random	1.30 (1.08, 1.56)	0.0051
Hispanic (2)	33 (6.00)	17 (4.43)	Fixed	1.34 (0.76, 2.34)	0.3044
East Asian (18)	79 (2.60)	26 (0.74)	Fixed	2.17 (1.46, 3.25)	0.0001
South Asian (6)	40 (4.16)	18 (2.08)	Fixed	2.11 (1.21, 3.65)	0.0076
Middle East (4)	65 (9.31)	25 (4.26)	Fixed	2.35 (1.47, 3.74)	0.0003
African (6)	104 (10.54)	80 (8.06)	Random	1.45 (0.87, 2.41)	0.1457
GT (61)	6066 (37.40)	3971 (32.49)	Random	1.37 (1.18, 1.57)	0.0001
Caucasian (25)	4271 (42.71)	2538 (43.69)	Random	0.96 (0.91, 1.02)	0.2571
Hispanic (2)	175 (31.81)	126 (32.81)	Fixed	0.96 (0.79, 1.16)	0.6801
East Asian (18)	672 (22.12)	518 (14.66)	Random	1.38 (1.19, 1.59)	0.0001
South Asian (6)	286 (29.79)	229 (26.44)	Fixed	1.17 (1.01, 1.36)	0.0304
Middle East (4)	272 (38.96)	180 (30.61)	Random	1.25 (0.92, 1.70)	0.1388
African (6)	390 (39.55)	380 (38.27)	Random	1.02 (0.83, 1.26)	0.8032
GG (61)	8613 (53.10)	7442 (60.89)	Random	0.92 (0.89, 0.95)	0.0001
Caucasian (25)	4498 (44.98)	2677 (46.09)	Random	0.95 (0.89, 1.01)	0.139
Hispanic (2)	342 (62.18)	241 (62.76)	Fixed	0.99 (0.89, 1.10)	0.9341
East Asian (18)	2286 (75.27)	2989 (84.60)	Random	0.91 (0.87, 0.95)	0.0001
South Asian (6)	634 (66.04)	619 (71.48)	Random	0.97 (0.84, 1.13)	0.7656
Middle East (4)	361 (51.72)	383 (65.14)	Fixed	0.77 (0.70, 0.85)	0.0001
African (6)	492 (49.90)	533 (53.68)	Random	0.91 (0.76, 1.07)	0.2809
TT + GT (61)	7617 (46.96)	4730 (38.70)	Random	1.15 (1.09, 1.22)	0.0001
Caucasian (25)	5501 (55.01)	3131 (53.90)	Random	1.03 (0.98, 1.09)	0.176
Hispanic (2)	208 (37.81)	143 (37.24)	Fixed	1.00 (0.85, 1.19)	0.9339
East Asian (18)	751 (24.73)	544 (15.40)	Random	1.44 (1.26, 1.64)	0.0001
South Asian (6)	326 (33.96)	247 (28.52)	Fixed	1.24 (1.08, 1.43)	0.0018
Middle East (4)	337 (48.28)	205 (34.86)	Fixed	1.42 (1.24, 1.63)	0.0001
African (6)	494 (50.10)	460 (46.32)	Random	1.10 (0.92, 1.33)	0.2638
T allele (61)	4585 (28.20)	2744 (22.5)	Random	1.18 (1.11, 1.25)	0.0001
Caucasian (25)	3366 (33.66)	1862 (32.05)	Random	1.07 (1.00, 1.16)	0.0466
Hispanic (2)	120 (21.82)	80 (20.83)	Fixed	1.04 (0.81, 1.34)	0.7399
East Asian (18)	415 (13.66)	285 (8.06)	Fixed	1.48 (1.28, 1.71)	0.0001
South Asian (6)	183 (19.06)	132 (15.24)	Fixed	1.30 (1.06, 1.60)	0.0106
Middle East (4)	201 (28.80)	115 (19.56)	Fixed	1.52 (1.24, 1.87)	0.0001
African (6)	299 (30.32)	270 (27.19)	Fixed	1.10 (0.95, 1.26)	0.1743
G allele (61)	11646 (71.80)	9428 (77.2)	Random	0.95 (0.93, 0.97)	0.0001
Caucasian (25)	6634 (66.34)	3946 (67.94)	Random	0.96 (0.92,0.99)	0.0304
Hispanic (2)	429 (78.00)	304 (79.17)	Fixed	0.98 (0.92, 1.05)	0.7404
East Asian (18)	2622 (86.34)	3248 (91.93)	Fixed	0.95 (0.93, 0.97)	0.0001
South Asian (6)	777 (80.94)	734 (84.76)	Random	1.02 (0.89, 1.16)	0.7493
Middle East (4)	497 (71.20)	473 (80.44)	Fixed	0.87 (0.82, 0.93)	0.0001
African (6)	687 (69.68)	723 (72.81)	Fixed	0.96 (0.90, 1.01)	0.1761

Note: CI = confidence interval; RR = risk ratio. Random effects models were used when *Q* or *I*^2^ were significant; otherwise, fixed effects models were used.

## Supplementary Materials

The following are available online at http://www.mdpi.com/2305-6304/6/3/44/s1, Table S1: Characteristics of included studies on *NOS3* 894 Genotypes, Table S2: Pooled meta-analysis: *NOS3* G894T genotypes and risk for ischemic heart disease (IHD) by subgroups, Figure S1: Ranked % *NOS3* homozygous TT and heterozygous GT for groups of control (*n* = 12,222) and ischemic heart disease (*n* = 16,219) from 61 study groups per country-race (number of study groups) and sample size, Figure S2: Forest plot for the meta-analysis of the associations between the *NOS3* TT genotype and ischemic heart disease (IHD) for RR > 1–2, Figure S3: Forest plot for the meta-analysis of the associations between the *NOS3* TT genotype and ischemic heart disease (IHD) for RR < 1, Figure S4: Geographical information map for % *NOS3* GT and TT genotypes per control and ischemic heart disease (IHD) groups, and IHD risk, Figure S5: Geographical information map for % *NOS3* GT genotype per control and ischemic heart disease (IHD) groups, and IHD risk, Figure S6: Nonlinear fit of *NOS3* G894T TT with death from air pollution for (a) all countries and (b) countries with pooled RR > 2.
