# Supplementary material for: Meta-Analysis of NOS3 G894T Polymorphisms with Air Pollution on the Risk of Ischemic Heart Disease Worldwide
Source: Toxics. 2018 Aug 1;6(3):44. doi: 10.3390/toxics6030044 (PMC6161281; doi:10.3390/toxics6030044)
Supplement: Supplementary file 1 [file toxics-06-00044-s001.pdf]

Toxics **2018**, *6*, 44; doi:10.3390/toxics6030044 www.mdpi.com/journal/toxics

Supplementary Materials: Meta-analysis of *NOS3 G894T* Polymorphisms with Air Pollution on the Risk of Ischemic Heart Disease Worldwide

Robin Johns, Zhao-Feng Chen, Lufei Young, Flordelis Delacruz, Nein-Tzu Chang, Chong Ho Yu and S. **Pamela K. Shiao**

Table S1. Characteristics of included studies on *NOS3 G894T* genotypes.

Author, Year	Country State or City	Ethnicity	Case, *n* GG	GT	TT	Controls, *n* GG	GT	TT	SC[a]	HWE	Quality Score[b]
**Caucasian**											
Granath 2001 [1]	Australia, Perth	Australian	260	248	63	270	287	66	2	Yes	22
Cai 1999 [2]	Australia, Sydney	Australian	286	249	70	66	70	22	2	Yes	13
Dosenko 2006 [3]	Ukraine, Kiev	Ukrainian	76	128	17	24	56	3	1	No	17
Bor-Kucukatay 2010 [4]	Turkey, Denizli	Turkish	48	28	7	50	21	3	2	Yes	17
Alp 2009 [5]	Turkey, Chongqing	Turkish	76	59	11	71	40	11	2	Yes	21
Berdeli 2005 [6]	Turkey, Izmir	Turkish	44	37	34	57	24	2	1	Yes	20
Afrasyap 2004 [7]	Turkey, Mugla	Turkish	114	103	33	74	62	14	1	Yes	21
Aras 2002 [8]	Turkey, Ankara	Turkish	89	87	29	60	48	9	1	Yes	9
Zigra 2013 [9]	Greece, Athens	Greek	43	38	7	50	42	11	1	Yes	19
Dafni 2010 [10]	Greece, Athens	Greek	83	94	27	108	95	15	1	Yes	23
Ragia 2010 [11]	Greece, Alexandroupolis	Greek	74	63	17	72	66	17	2	Yes	22
Andrikopoulos 2008 [12]	Greece, Athens	Greek	722	702	179	352	297	78	2	Yes	19
Vasilakou 2008 [13]	Greece, Athens	Greek	109	85	15	76	74	11	2	Yes	15
Fatini 2004 [14]	Italy, Florence	Italian	185	213	79	236	243	58	1	Yes	15
Colombo 2002 [15]	Italy, Massa	Italian	91	78	32	48	59	7	2	Yes	18
Agema 2004 [16]	Netherlands, Amsterdam,	Dutch	343	333	79	216	270	88	2	Yes	24
Pulkinnen 2000 [17]	Finland, Koupio	Finnish	263	237	59	54	45	11	1	Yes	24
Gluba 2009 [18]	Poland, Lodz	Polish	140	118	19	62	61	11	2	Yes	18
Szperl 2008 [19]	Poland, Warsaw	Polish	69	67	15	39	28	7	1	Yes	22
Gardemann, 2002 [20]	Germany, Giessen	German	924	927	234	256	227	50	1	Yes	19
Poirier 1999 [21]	Ireland-Belfast France-Toulouse Strasbourg, Lille	N. Irish, French	163	156	49	148	219	54	2	Yes	22
Bhandary 2008 [22]	United Kingdom, Plymouth	British	51	91	27	73	51	17	2	Yes	20
Hingorani 1999 [23]	United Kingdom, Cambridge	British	120	71	107	66	58	14	1	Yes	25
Piccoli 2012 [24]	Brazil, Porto Alegre	Brazilian	58	52	18	62	44	7	2	Yes	23
Jaramillo 2010 [25]	Chile, Temuco	Chilean	73	31	8	74	36	2	1	Yes	22
**Hispanic**											
García-González 2015 [26]	Mexico, Merida	Mexican	62	28	8	68	31	2	2	Yes	22
Vargas-Alarcon 2014 [27]	Mexico, Mexico City	Mexican	280	147	25	173	95	15	1	Yes	18
**East Asian**											
Hibi 1998 [28]	Japan	Kanagawa	189	32	0	295	62	0	2	Yes	19
Shimasaki 1998 [29]	Japan, Kyoto Kumamoto	Japanese	225	59	1	526	80	1	2	Yes	24
Bae 2010 [30]	South Korea, Jeju	Korean	157	34	1	163	33	0	1	Yes	20
Min 2010 [31]	South Korea, Gwangju	Korean	136	28	4	250	20	0	2	Yes	22
Kim 2007 [32]	South Korea, Seongnam	Korean	119	28	0	181	38	0	1	Yes	24
Park 2004 [33]	South Korea, Seoul	Korean	128	35	1	118	22	2	2	Yes	21
Yoon 2000 [34]	South Korea, Chongno-gu	Korean	94	15	1	110	18	0	1	Yes	24
Wang 2001 [35]	Taiwan, Taipei	Chinese	178	38	2	177	38	3	2	Yes	25
Lin 2008 [36]	China, Taiwan	Chinese	25	89	6	49	23	6	2	Yes	24

Table S1. *Cont.*

**Author, Year**	**Country State or City**	**Ethnicity**	**Case, *n***			**Controls, *n***			**SC[a]**	**HWE**	**Quality Score[b]**
			**GG**	**GT**	**TT**	**GG**	**GT**	**TT**			
Wang* 2011 [37]	China, Louyang	Chinese	151	46	6	163	29	2	2	Yes	23
Cui 2011 [38]	China, Henan	Chinese	125	20	3	99	14	2	1	Yes	21
Huang De 2010 [39]	China, Gansu	Chinese	151	46	6	163	29	2	2	Yes	19
Yusup 2009 [40]	China, Xinjiang	Chinese	35	64	25	24	20	6	1	Yes	20
Jinyu 2009 [41]	China, Changsha	Chinese	77	22	4	89	14	1	2	Yes	22
Ying 2008 [42]	China	Chinese	174	30	3	237	26	1	2	Yes	20
Huang 2008 [43]	China, Gansu	Chinese	152	39	5	162	27	0	2	Yes	21
Qi 2003 [44]	China, ShenYang city	Chinese	86	28	3	85	15	0	2	Yes	20
Wei 2002 [45]	China, Hangzhou	Chinese	84	19	3	98	10	0	1	Yes	23
**South Asian**											
Arun Kumar 2013 [46]	India, Pondicherry	Indian	213	62	12	238	76	7	1	Yes	16
Narne 2013 [47]	India, Hyderabad	Indian	97	60	3	84	35	2	2	Yes	20
Rai 2012 [48]	India, Pradesh	Indian	159	84	10	119	50	5	1	Yes	21
Saini 2011 [49]	India, New Delhi	Indian	45	15	0	44	6	0	1	Yes	21
Bhanushali 2010 [50]	India, Mumbai	Indian	64	35	1	67	31	2	2	Yes	18
Angeline 2010 [51]	India, Tamil Nadu	Indian	56	30	14	67	31	2	1	Yes	19
**Middle East**											
Rahimi 2012 [52]	Iran, Kermanshah	Iranian	116	74	17	67	24	1	2	Yes	20
Salimi 2010 [53]	Iran, Zahedan	Iranian	112	103	26	160	84	17	2	Yes	20
Heidari 2016 [54]	Iran, Yazd	Iranian	68	28	12	58	32	0	1	Yes	18
Alkharfy 2010 [55]	Saudi Arabia, Riyadh	Saudi	65	67	10	98	40	7	2	Yes	22
**African**											
Abdel-Aziz 2013 [56]	Egypt, Zagazig	Egyptian	48	46	22	68	39	12	2	Yes	18
Motawi 2011 [57]	Egypt, Cairo	Egyptian	46	34	20	19	28	3	2	Yes	21
Kallel 2013 [58]	Tunisia, Tunis	Tunisian	163	122	18	128	78	19	2	Yes	20
Abdelhedi 2013 [59]	Tunisia, Tunisia	Tunisian	130	94	25	130	130	35	2	Yes	21
Kerkene 2006 [60]	Tunisia, Monastir	Tunisian	45	44	11	72	43	5	1	Yes	22
Idrissi 2016 [61]	Morocco, Casablanca	Morocco	60	50	8	116	62	6	1	Yes	21

Note. [a]SC: Sources of control: 1 = healthy adults, 2 = adults without cardiovascular disease. HWE = Hardy Weinberg Equilibrium (HWE). [b]Quality Score ranges: 0–30. * = meta-analysis paper + case control study.

Table S2. Pooled meta-analysis: *NOS3* G894T genotypes and risk for ischemic heart disease (IHD) by subgroups.

Genotype. (Number of Studies)	Cases n = 13,207 (%)	Controls n = 10,021 (%)	Model	Tests of Association RR (95% CI)	p
Subgroups					
TT risk > 1–2					
TT (29)	921 (6.97)	398 (3.97)	Random	1.62 (1.38, 1.90)	< 0.0001
GT (29)	3,590 (27.18)	1,973 (19.78)	Random	1.10 (1.03, 1.19)	0.0039
GG (29)	4,674 (35.39)	3,397 (33.89)	Random	0.91 (0.88, 0.94)	< 0.0001
TT + GT (29)	4,511 (34.15)	2,371 (23.66)	Random	1.19 (1.13, 1.27)	< 0.0001
TT risk > 2					
TT (24)	330 (2.49)	108 (1.07)	Fixed	2.44 (1.96, 3.02)	< 0.0001
GT (24)	1201 (9.09)	1031 (10.28)	Random	1.09 (0.96, 1.25)	0.1667
GG (24)	2503 (18.95)	3064 (30.57)	Random	0.92 (0.87, 0.97)	0.0017
TT + GT (24)	1531 (11.59)	1139 (11.36)	Random	1.22 (1.12, 1.33)	< 0.0001
TT risk < 1					
TT (8)	300 (2.27)	253 (2.52)	Fixed	0.85 (0.72, 1.00)	0.053
GT (8)	1275 (9.65)	967 (9.64)	Fixed	0.96 (0.90, 1.02)	0.2863
GG (8)	1436 (10.87)	981 (9.78)	Fixed	1.04 (0.94, 1.15)	0.3904
TT + GT (8)	1575 (11.92)	1220 (12.17)	Random	0.95 (0.88, 1.04)	0.3086
TT + GT risk > 1–2					
TT (48)	1,143 (8.65)	471 (4.70)	Random	1.54 (1.30, 1.83)	< 0.0001
GT (48)	4,356 (32.98)	2,678 (26.72)	Random	1.13 (1.05, 1.22)	0.0007
GG (48)	6,565 (49.70)	6,019 (60.06)	Random	0.90 (0.87, 0.94)	< 0.0001
TT + GT (48)	5,499 (41.63)	3,149 (31.42)	Random	1.21 (1.14, 1.28)	< 0.0001
TT + GT risk < 1					
TT (8)	333 (2.52)	251 (2.50)	Random	1.11 (0.79, 1.55)	0.5281
GT (8)	1,319 (9.98)	1,042 (10.39)	Random	0.95 (0.85, 1.06)	0.3661
GG (8)	1,424 (10.78)	1,009 (10.06)	Random	1.01 (0.88, 1.17)	0.8014
TT + GT (8)	1,652 (12.50)	1,293 (12.90)	Random	0.99 (0.88, 1.11)	0.9328
TT + GT risk mixed					
TT (5)	75 (0.56)	37 (0.03)	Fixed	1.22 (0.84, 1.78)	0.279
GT (5)	391 (2.96)	251 (2.50)	Fixed	0.96 (0.84, 1.09)	0.5728
GG (5)	624 (4.72)	414 (4.13)	Fixed	0.99 (0.92, 1.08)	0.9859
TT + GT (5)	466 (3.52)	288 (2.87)	Fixed	0.99 (0.89, 1.11)	0.9881

Note. CI = confidence interval; RR = risk ratio. Random effects models were used when Q or I^2 were significant; otherwise, fixed effects models were used. *TT* risk > 1–2, nine countries: Turkey, Greece, Italy, Finland, Germany, Ireland, Mexico, China, Saudi Arabia; *TT* risk > 2, 10 countries: Ukraine, the United Kingdom, Brazil, Chile, Japan, South Korea, India, Iran, Egypt, Morocco; *TT* risk < 1, four countries: Australia, the Netherlands, Poland, Tunisia; *TT* risk mixed, None. *TT+GT* risk > 1–2, 15 countries: Turkey, Greece, Italy, Finland, Germany, the United Kingdom, Brazil, Korea, China, India, Saudi Arabia, Egypt, Tunisia, Morocco; *TT*+GT risk > 2, None; *TT+GT* risk < 1, five countries: Australia, Ukraine, the Netherlands, Ireland, Iran; *TT+GT* risk mixed, Poland, Chile, Mexico.

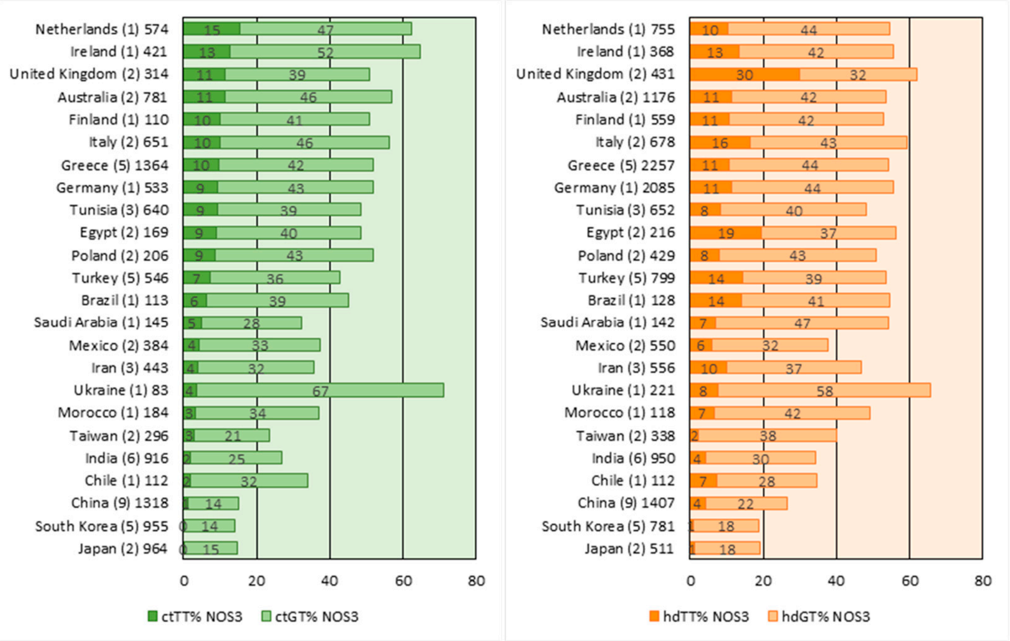

Figure S1. Ranked % *NOS3* homozygous TT and heterozygous GT for groups of control (n = 12,222) and ischemic heart disease (n = 16,219) from 61 study groups per country-race (number of study groups) and sample size.

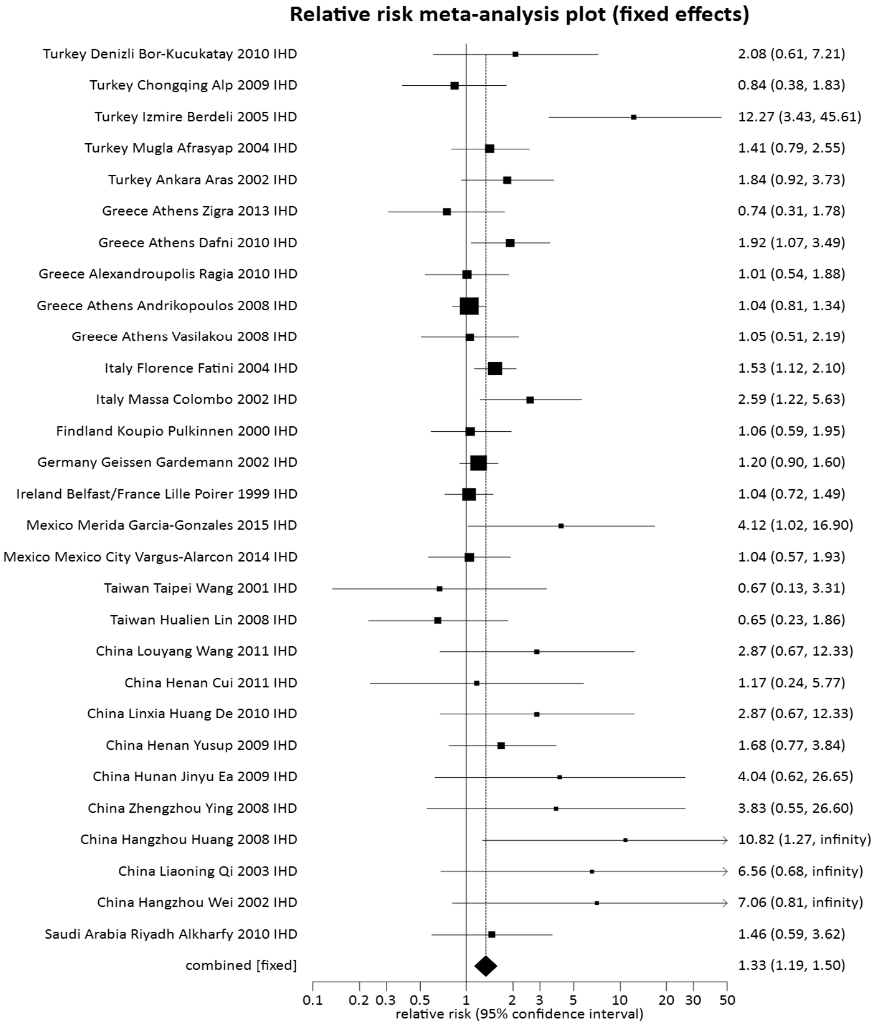

Figure S2. Forest plot for countries with pooled RR > 1–2 for the meta-analysis of the associations between *NOS3* TT genotype and ischemic heart disease (IHD). The midpoint = point effect estimate for each study. The area of the box = weight given to the study. The length of the line = 95% confidence interval for the effect estimate in an individual study. The width of the diamond = 95% confidence interval for the overall effect estimate.

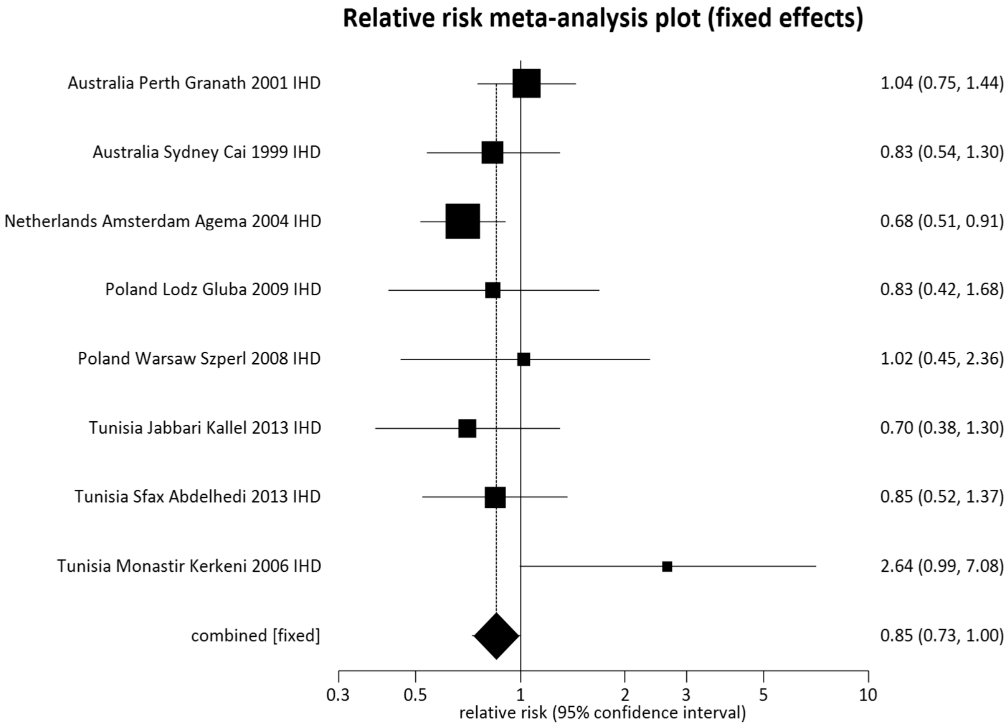

Figure S3. Forest plots for countries with pooled RR < 1 for the meta-analysis of the associations between *NOS3* TT genotypes and ischemic heart disease (IHD). The midpoint = point effect estimate for each study. The area of the box = weight given to the study. The length of the line = 95% confidence interval for the effect estimate in an individual study. The width of the diamond = 95% confidence interval for the overall effect estimate.

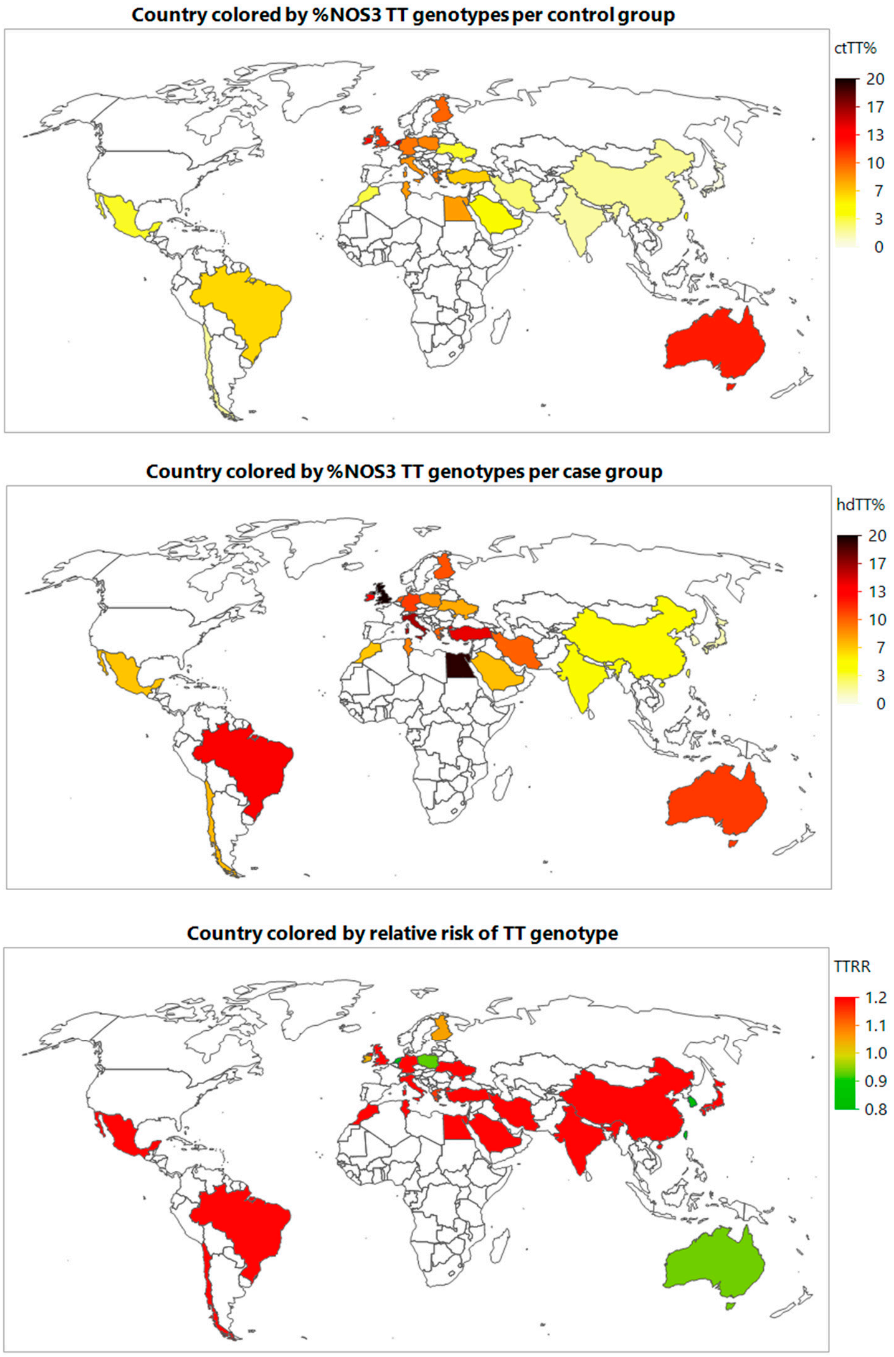

Figure S4. Geographical information map for % *NOS3* TT genotype per control and ischemic heart disease (IHD) groups, and IHD risk.

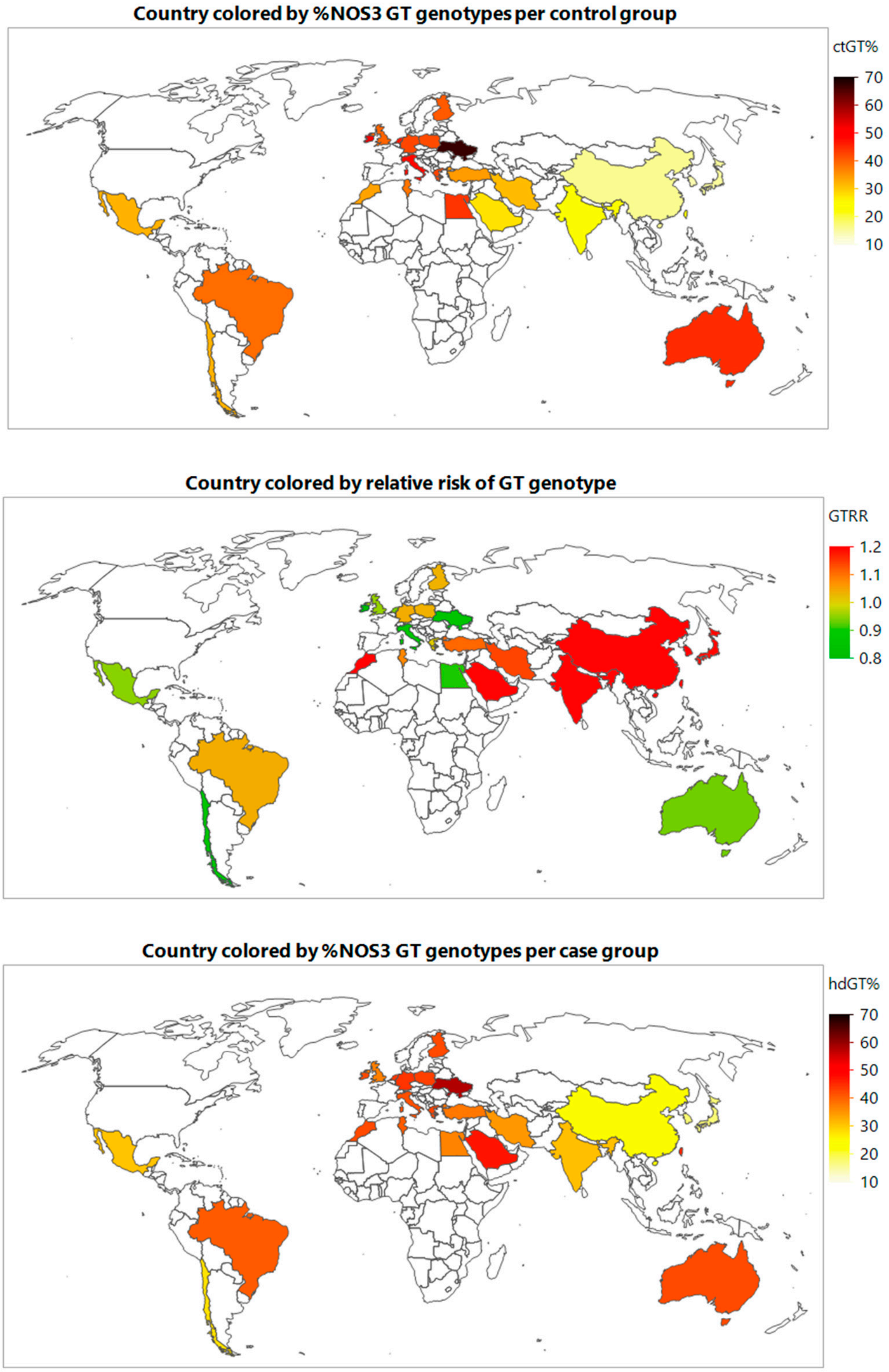

Figure S5. Geographical information map for % *NOS3* GT genotype per control and ischemic heart disease (IHD) groups, and IHD risk.

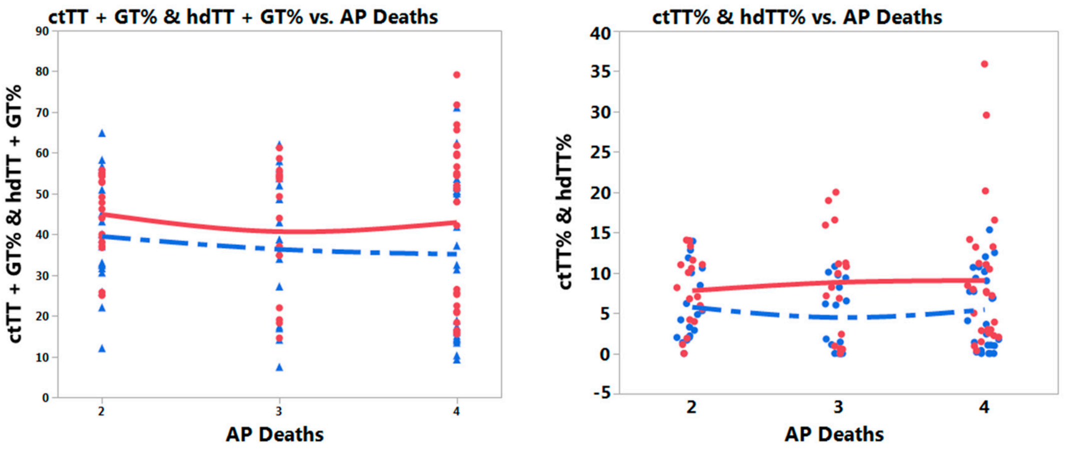

(a) (b)

Figure S6. Nonlinear fit of *NOS3* G894T polymorphisms with death from air pollution for all countries: (**a**) TT plus GT polymorphisms, (**b**) TT polymorphism only (AP death: Death rates from air pollution, Levels per million: 2 = 51–100, 3 = 101–250, 4 = 251–400 and greater); case = red (solid line), control = blue (dotted line).

References

1. Granath, B.; Taylor, R.R.; van Bockxmeer, F.M.; Mamotte, C.D. Lack of evidence for association between endothelial nitric oxide synthase gene polymorphisms and coronary artery disease in the Australian Caucasian population. *J. Cardiovasc. Risk* ***2001***, *8*, 235–241, doi:10.1177/174182670100800408.
2. Cai, H.; Wilcken, D.E.; Wang, X.L. The Glu-298–>Asp (894G–>T) mutation at exon 7 of the endothelial nitric oxide synthase gene and coronary artery disease. *J. Mol. Med.* **1999**, *77*, 511–514, doi:10.1007//s001099900020.
3. Dosenko, V.E.; Zagoriy, V.Y.; Lutay, Y.M.; Parkhomenko, A.N.; Moibenko, A.A. Allelic polymorphism in the promoter (T-786–>C), but not in exon 7 (G894–>T) or the variable number tandem repeat in intron 4, of the endothelial nitric oxide synthase gene is positively associated with acute coronary syndrome in the Ukrainian population. *Exp. Clin. Cardiol.* **2006**,*11*, 11–13.
4. Bor-Kucukatay, M.; Demir, S.; Akbay, R.; Dursunoglu, D.; Akdag, B.; Semiz, E. Eelationship between hemorheology and Glu(298)Asp polymorphism of endothelial nitric oxide synthase gene in patients with coronary artery disease. *Mol. Biol. Rep.* **2010**, *37*, 171–178, doi:10.1007/s11033-009-9572-9.
5. Alp, E.; Menevse, S.; Tulmac, M.; Kan, D.; Yalcin, R.; Erkan, A.F.; Cengel, A. Lack of association between matrix metalloproteinase-9 and endothelial nitric oxide synthase gene polymorphisms and coronary artery disease in Turkish population. *DNA Cell Biol.* **2009**, *2*, 343–350, doi:10.1089/dna.2009.0866.
6. Berdelli, A.; Sekuri, C.; Cam, F.; Ercan, E.; Sagcan, A.; Tengiz, I.; Akin, M. Association between the *eNOS* (Glu298Asp) and the RAS genes polymorphisms and premature coronary artery disease in a Turkish population. *Clin. Chim. Acta* **2005**, *351*, 87–94, doi:10.1016/j.cccn.2004.08.015.
7. Afrasyap, L.; Ozturk, G. NO level and endothelial NO synthase gene polymorphism (Glu298Asp) in the patients with coronary artery disease from the Turkish population. *Acta Bioch. Bioph. Sin.* **2004**, *36*, 661–666.
8. Aras, O.; Hanson, N.Q.; Bakanay, S.M.; Tsai, M.Y.; Gulec, S. Endothelial nitric oxide gene polymorphism (Glu298Asp) is not associated with coronary artery disease in Turkish population. *Thromb. Haemost.* **2002**, *87*, 347–349.
9. Zigra, A.M.; Rallidis, L.S.; Anatasiou, G.; Merkouri, E.; Gialeraki, A. eNOS gene variants and the risk of premature myocardial infarction. *Dis. Markers* **2013**, *34*, 431–436, doi:10.3233/DMA-130987.
10. Dafni, C.; Drakoulis, N.; Landt, O.; Panidis, D.; Reczko, M; Cokkinos, D.V. Association of the eNOS E298D polymorphism and the risk of myocardial infarction in the Greek population. *BMC Med. Genet.* **2010**, *11*, 133, doi:10.1186/1471-2350-11-133.
11. Ragia, G.; Nikolaidis, E.; Tavridou, A.; Arvanitidis, K.I.; Kanoni, S.; Dedoussis, G.V.; Manolopoulos, V.G. Endothelial nitric oxide synthase gene polymorphisms -786T > C and 894G > T in coronary artery bypass graft surgery patients. *Hum. Genom.* **2010**, *4*, 375–383.

12. Andrikopoulos, G.K.; Grammatopoulos, D.K.; Tzeis, S.E.; Zervou, S.; Richter, D.J.; Zairis, M.N.; Hillhouse, E.W. Association of the 894G > T polymorphism in the endothelial nitric oxide synthase gene with risk of acute myocardial infarction. *BMC Med. Genet.* **2008**, *9*, 1–6, doi:10.1186/1471-2350-9-43.
13. Vasilakou, M.; Votteas, V.; Kasparian, C.; Pantazopoulos, N.; Dedoussis, G.; Deltas, C.; Lamnissou, K. Lack of association between nitric oxide synthase gene polymorphism and risk of premature coronary artery disease in the Greek population. *Acta Cardiol.* **2008**, *63*, 609–614, doi:10.2143/AC.63.5.2033229.
14. Fatini, C.; Sofi, F.; Sticchi, E.; Gensini, F.; Gori, A.M.; Fedi, S.; Abbate, R.I. Influence of endothelial nitric oxide synthase gene polymorphisms (G894T, 4a4b, T-786C) and hyperhomocysteinemia on the predisposition to acute coronary syndromes. *Am. Heart J.* **2004**, *147*, 516–521.
15. Colombo, M.G.; Andreassi; M.G.; Paradossi, U.; Botto, N.; Manfredi, S.; Masetti, S.; Biagini, A. Evidence for association of a common variant of the endothelial nitric oxide synthase gene (Glu298Asp polymorphism) to the presence, extent, and severity of coronary artery disease. *Heart* **2002**, *82*, 525–528.
16. Agema. W.R.; De Maat, M.P.; Zwinderman, A.H.; Kastelein, J.J.; Rabelink, T.J.; Rabelink, T.J.; Jukema, J.W. An integrated evaluation of endothelial constitutive nitric oxide synthase polymorphisms and coronary artery disease in men. *Clin. Sci.* **2004**, *107*, 255–261.
17. Pulkkinen, A.; Viitanen, L.; Kareinen, A.; Lehto, S.; Vauhkonen, I.; Laakso, M. Intron 4 polymorphism of the endothelial nitric oxide synthase gene is associated with elevated blood pressure in type 2 diabetic patients with coronary heart disease. *J. Mol. Med.* **2000**, *78*, 372–379, doi:10.1007/s001090000124.
18. Gluba, A.; Banach, M; Rysz, J; Piotrowski, G; Fendler, W; Pietrucha, T. Is polymorphism within eNOS gene associated with the late onset of myocardial infarction? A pilot study. *Angiology* **2009**, *60*, 588–595, doi:10.1177/0003319709335031.
19. Szperl, M.; Dzielinska, Z.; Roszczynko, M.; Malek, L.A.; Makowiecka-Ciesla, M.; Demkow, M.; Ruzyllo, W. Genetic variants in hypertensive patients with coronary artery disease and coexisting atheromatous renal artery stenosis. *Med. Sci. Monit.* **2008**, *14*, CR611–CR616.
20. Gardemann. A.; Lohre, J.; Cayci, S.; Katz, N.; Tillmanns, H.; Haberbosch, W. The T allele of the missense Glu(298)Asp endothelial nitric oxide synthase gene polymorphism is associated with coronary heart disease in younger individuals with high atherosclerotic risk profile. *Atherosclerosis* **2002**, *160*, 167–175, doi:10.1016/S0021-9150(01)00554-8.
21. Poirier, O.; Mao, C.; Mallet, C.; Nicaud, V.; Herrmann, S.M.; Evans, A.; Ruidavets. J.B.; Cambien, F. Polymorphisms of the endothelial nitric oxide synthase gene - no consistent association with myocardial infarction in the ECTIM study. *Eur. J. Clin. Investig.* **1999**, *29*, 284–290.
22. Bhandary, U.V.; Tse, W.; Yang, B.; Knowles, M.R.; Demaine, A.G. Endothelial nitric oxide synthase polymorphisms are associated with hypertension and cardiovascular disease in renal transplantation. *Nephrology* **2008**, *13*, 348–355, doi:10.1111/j.1440-1797.2008.00925.
23. Hingorani, A.D.; Liang, C.F.; Fatibene, J.; Lyon, A; Monteith, S.; Parson, A; Brown, M.J. A common variant of the endothelial nitric oxide synthase (Glu298–>Asp) is a major risk factor for coronary artery disease in the UK. *Circulation* ***1999***, *100*, 1515–1520, doi:10.1161/01.CIR.100.14.1515.
24. Da Costa Escobar Piccoli, J.; Manfredini. V.; Hamester, F.I.; Bandinelli. J.B.; Turkienicz, I.M.; Chies, J.A.; Peres A.; Bogo, M.R. Interaction between endothelial nitric oxide synthase gene polymorphisms (-786T>C, 894G>T and intron 4 a/b) and cardiovascular risk factors in acute coronary syndromes. *Arch. Med. Res.* ***2012***, *43*, 205–211, doi:10.1016/j.arcmed.2012.03.011.
25. Jaramillo, P.C.; Lanas, C.; Lanas, F.; Salazar, L.A. Polymorphisms of the NOS3 gene in Southern Chilean subjects with coronary artery disease and controls. *Clin. Chim. Acta* **2010**, *411*, 258–262, doi:10.1016/j.cca.2009.11.018.
26. García-González, I.; Solís-Cárdenas Ade, J.; Flores-Ocampo, J.A.; Alejos-Mex R.; Herrera-Sánchez, L.F.; González-Herrera, L.J. G894T (NOS3) and G1958A (MTHFD1) gene polymorphisms and risk of ischemic heart disease in Yucatan, Mexico. *Clin. Investig. Arterioscler.* **2015**, *27*, 64–73, doi:10.1016/j.arteri.2014.07.002.
27. Vargas-Alarcon, G.; Vallejo, M.; Posadas-Romero, C.; Juarez-Rojas, J.G.; Martinez-Rios, M.A.; Peña-Duque, M.A.; Carrillo-Sanchez, S.; Perez-Mendez, O.; Fragoso, J.M. The -974C>A (rs3087459) gene polymorphism in the endothelin gene (EDN1) is associated with risk of developing acute coronary syndrome in Mexican patients. *Gene* ***2014***, *542*, 258–262, doi:10.1016/j.gene.2013.09.003.

28. Hibi, K.; Ishigami, T.; Tamura, K.; Mizushima, S.; Nyui, N.; Fujita, T.; Umemura, S. Endothelial nitric oxide synthase gene polymorphism and acute myocardial infarction. *Hypertension* ***1998***, *32*, 521–526, doi:10.1161/01.HYP.32.3.52.
29. Shimasaki, Y.; Yasue, H.; Yoshimura, M.; Nakayama, M.; Kugiyama, K.; Ogawa, H.; Nakao, K. Association of the missense Glu298Asp variant of the endothelial nitric oxide synthase gene with myocardial infarction. *J. Am. Coll. Cardiol*. **1998**, *31*, 1506–1510, doi:10.1016/S0735-1097(98)00167-3.
30. Bae, J.; Kim, I.J.; Hong, S.H.; Sung, J.H.; Lim, S.W.; Cha, D.H.; Cho, Y.W.; Oh, D.; Kim, N.K. Association of endothelial nitric oxide synthase polymorphisms with coronary artery disease in Korean individuals with or without diabetes mellitus. *Exp. Ther. Med.* **2010**, *1*, 719–724.
31. Min, B.W.; Na, J.Y.; Juhng, S.W.; Park, M.S.; Park, J.T. A polymorphism (G894T) in eNOS increases the risk of coronary atherosclerosis rather than intracranial atherosclerosis in Koreans. *Acta Neurol. Belg.* **2010**, *110*, 255–262.
32. Kim, I.J.; Bae, J.; Lim, S.W.; Cha, D.H.; Cho, H.J.; Kim, S.; Kim, N.K. Influence of endothelial nitric oxide synthase gene polymorphisms (−786T-C, 4a4b, 894G-T) in Korean patients with coronary artery disease. *Thromb. Res.* **2007**, *119*, 579–585.
33. Park, K.W.; You, K.H.; Oh, S.; Chae, I.H.; Kim, H.S.; Oh, B.H.; Park, Y.B. Association of endothelial constitutive nitric oxide synthase gene polymorphism with acute coronary syndrome in Koreans. *Heart* **2004**, *90*, 282–285.
34. Yoon, Y.; Song, J.; Hong, S.H.; Kim, J.Q. Plasma nitric oxide concentrations and nitric oxide synthase gene polymorphisms in coronary artery disease. *Clin. Chem.* **2000**, *46*, 1626–1630.
35. Wang, C.L.; Hsu, L.A.; Ko, Y.S.; Ko, Y.L.; Lee, Y.H. Lack of association between the Glu298Asp variant of the endothelial nitric oxide synthase gene and the risk of coronary artery disease among Taiwanese. *J. Formos. Med. Assoc.* **2001**, *100*, 736–740.
36. Lin, N.; Lee, M.; Lee, R.; Hong, A.; Chen, H. Analysis of endothelial nitric oxide synthase gene polymorphisms with cardiovascular diseases in eastern Taiwan. *Chin. J. Physiol.* **2008**, *51*, 42–47.
37. Wang, H.Z.; Dong, P. The correlation between endothelial nitric oxide synthase gene G894T polymorphism and coronary heart disease. *Clin. Ration Drug Use* **2011**, *4*, 32–34.
38. Cui, Q.T.; Hp, L.; Fu, Q.L.; Jie,Z. Association analysis of eNOS gene polymorphisms and coronary artery disease. *Chin. J. Clin.* **2011**, *5*, 46–49.
39. Huang De, Z.M.J.Y.; Zhang, Z.F.; Ma, G.R.; Yang, F.; Min, H. Correlation of endothelial nitric oxide synthase gene G894T polymorphism with coronary heart disease on Dongxiang ethnic minority. *Gansu Med. J.* **2010**, *29*, 374–377.
40. Yusup, A.; Ibrayim, A.; Upur, H. The gene polymorphism of ACE, eNOS, FVII and ICAM-1 genes in uighur patients with coronary heart disease in Xinjiang. *Sci. Tech. Rev.* **2009**, 27, 76–81.
41. Jinyu, P. Relationship between coronary heart disease and polymorphisms of endothelial nitric oxide synthase gene G894T in Han Nationality. *J. Chongqing Med. Univ.* **2009**, *34*, 20–23.
42. Ying, H.E.; Yd, Z.; Hui, Y.U.; Zhao, L.S.; Zheng, H. Research on correlativity of 27 bpVNTR and G894T polymorphisms of endothelial nitric oxide synthase gene and coronary heart disease in Henan Han population. *Chin. J. Mod. Med.* ***2008***, *18*, 523–527.
43. Huang, X.G.X.; Jin, G. Relative study among the G894T polymorphism of endothelial nitric oxide synthase gene, smoking and coronary artery disease. *Clin. Educ. Gen. Pract.* **2008**, *16*, 207–210.
44. Qi, J.; Dai, C.; Huang, T.; Ma, Y.; Yuan, D.; Zeng, D. A smoking-dependent risk of coronary artery disease associated with a polymorphism of the endothelial nitric oxide synthase gene. *Chin. J. Clin. Rehabilit.* **2003**, *7*, 1476–1477.
45. Wei, D.; Shan, J.; Chen, Z.; Shi, Y. The G894T mutation of the endothelial nitric oxide synthase gene is associated with coronary atherosclerotic heart disease in Chinese. *Chin. J. Med. Genet.* **2002**, *19*, 471–474.
46. Arun Kumar, A.S.; Umamaheswaran, G.; Padmapriya, R.; Balachandar, J.; Adithan, C. Endothelial nitric oxide synthase gene polymorphisms and the risk of acute myocardial infarction in a South Indian population. *Mol. Biol. Rep.* **2013**, *40*, 1275–1281, doi:10.1007/s11033-012-2170-2.
47. Narne, P.; Ponnaluri, K.C.; Singh, S.; Siraj, M.; Ishaq, M. Association of the genetic variants of endothelial nitric oxide synthase gene with angiographically defined coronary artery disease and myocardial infarction in South Indian patients with type 2 diabetes mellitus. *J. Diabetes Complicat.* ***2013***, *27*, 255–261, doi:10.1016/j.jdiacomp.2012.10.009.

48. Rai, H.; Fitt, J.; Sharma, A.K.; Sinha, N.; Kumar, S.; Pandey, C.M., Agrawal, S.; Mastana, S. Lack of association between Glu298Asp polymorphism and coronary artery disease in North Indians. *Mol. Biol. Rep.* **2012**, *39*, 5995–6000, doi:10.1007/s11033-011-1412-z.
49. Saini, V.; Bhatnagar, M.K.; Bhattacharjee, J. Endothelial nitric oxide synthase Glu298Asp (G894T) gene polymorphism in coronary artery disease patients with type 2 diabetes mellitus. *Diabetes Metab. Syndr.* **2011**, 6, 106–109, doi:10.1016/j.dsx.2012.05.001.
50. Bhanushali, A.A.; Das, B.R. Genetic variants at the APOE, lipoprotein lipase (LpL), cholesteryl ester transfer protein (CETP), and endothelial nitric oxide (eNOS) genes and coronary artery disease (CAD): CETP Taq1 B2B2 associates with lower risk of CAD in Asian Indians. *J. Community Genet.* **2012**, *1*, 55–62.
51. Angeline, T.; Isabel, W.; Tsongalis, G.J. Endothelial nitric oxide gene polymorphisms, nitric oxide production and coronary artery disease risk in a South Indian population. *Exp. Mol. Pathol.* **2010**, *89*, 205–208, doi:10.1016/j.yexmp.2010.08.009.
52. Rahimi, Z.; Nourozi-Rad, R.; Rahimi, Z.; Parsian, A. Strong interaction between T allele of endothelial nitric oxide synthase with B1 allele of cholesteryl ester transfer protein TaqIB highly elevates the risk of coronary artery disease and type 2 diabetes mellitus. *Hum. Genom.* **2012**, *6*, 20, doi:10.1186/1479-7364-6-20.
53. Salimi, S.; Firoozrai, M.; Zand, H.; Nakhaee, A.; Shafiee, S.M., Tavilani, H.; Mohebbi, A. Endothelial nitric oxide synthase gene Glu298Asp polymorphism in patients with coronary artery disease. *Ann. Saudi Med.* **2010**, *30*, 33–37, doi:10.4103/0256-4947.59370.
54. Heidari, M.; Khatami, M.; Hadadzadeh, M.; Kazemi, M.; Mahamed, S.; Malekzadeh, P.; Mirjalili, M. Polymorphism in NOS3, MTHFR, APOB, and TNF-a genes and coronary atherosclerotic lesions in iranian patients. *Res. Cardiovasc. Med.* **2016**, *5*, e29134, doi:10.5812/cardiovascmed.29134.
55. Alkharfy, K.M.; Al-Daghri, N.M.; Al-Attas, O.S.; Alokail, M.S. Draz, H.M.; Hussain, T. Endothelial nitric oxide synthase gene polymorphisms (894G. T and -786T. C) and risk of coronary artery disease in a Saudi population. *Arch. Med. Res.* **2010**, *41*, 134–141.
56. Abdel-Aziz, T.A.; Mohamed, R.H. Association of endothelial nitric oxide synthase gene polymorphisms with classical risk factors in development of premature coronary artery disease. *Mol. Biol. Rep.* **2013**, *40*, 3065-3071, doi:10.1007/s11033-012-2380-7.
57. Motawi, T.; Shaker, O.; Taha, M.; Sedrak, H.; Nabil, M. Endothelial nitric oxide synthase and angiotensinogen gene polymorphism in coronary artery diseases in Egypt. *Angiology* **2011**, *62*, 191–197.
58. Kallel, A.; Sbai, M.H.; Sediri, Y.; Abdessalem, S.; Mourali, M.S.; Feki, M.; Mechmeche, R.; Jemaa R.; Kaabachi N. Polymorphisms of the NOS3 gene and risk of myocardial infarction in the Tunisian population. *Cytokine* **2013**, *64*, 646–651.
59. Abdelhedi, R.; Kharrat, N.; Bouayed, N.A.; Abid, L.; Abdelmouleh, W.; Sahnoun, I.T.; Alfadhli, S.; Laroussi, L.; Rebai, A. Lack of association of NOS3 and ACE gene polymorphisms with coronary artery disease in Southern Tunisia. *Biochem. Genet.* **2013**, *51*, 92–100, doi:10.1007/s10528-012-9545-x.
60. Kerkeni, M.; Addad, F.; Chauffert, M.; Myara, A.; Ben Farhat, M.; Miled, A.; Khira Maaroufi, K.; Trivin, F. Hyperhomocysteinemia, endothelial nitric oxide synthase polymorphism, and risk of coronary artery disease. *Clin. Chem.* **2006**, *52*, 53–58.
61. Idrissi, H.; Hmimech, W.; Diakete, B.; Korchi, F.; Baghdadi, D.; Habai, R.; Nadifi, S. Association of G894T eNOS, 4G/5G PAI and T1131C APOA5 polymorphisms with susceptibility to myocardial infarction in Morocco. *Meta Gene* **2016**, 9, 56–61, doi:10.1016/j.mgene.2016.03.004.
